# Association of Physical Activity Patterns With Nocturnal Hypoglycemia Events in Youth With Type 1 Diabetes

**DOI:** 10.1210/clinem/dgae451

**Published:** 2024-07-02

**Authors:** Ignacio Hormazábal-Aguayo, Nidia Huerta-Uribe, Jacinto Muñoz-Pardeza, Yasmin Ezzatvar, Mikel Izquierdo, Antonio García-Hermoso

**Affiliations:** Navarrabiomed, Complejo Hospitalario de Navarra, Universidad Pública de Navarra, IdiSNA, Pamplona 31008, Spain; Navarrabiomed, Complejo Hospitalario de Navarra, Universidad Pública de Navarra, IdiSNA, Pamplona 31008, Spain; Navarrabiomed, Complejo Hospitalario de Navarra, Universidad Pública de Navarra, IdiSNA, Pamplona 31008, Spain; Department of Nursing, Universitat de València, Valencia 46010, Spain; Navarrabiomed, Complejo Hospitalario de Navarra, Universidad Pública de Navarra, IdiSNA, Pamplona 31008, Spain; Navarrabiomed, Complejo Hospitalario de Navarra, Universidad Pública de Navarra, IdiSNA, Pamplona 31008, Spain

**Keywords:** glycemic control, exercise, accelerometer, insulin-dependent diabetes mellitus

## Abstract

**Aims:**

This study sought to elucidate the interactions among physical activity (PA) patterns, mean glucose concentrations, and the incidence of nocturnal hypoglycemia events in children and adolescents with type 1 diabetes, examining the moderating influence of daily dosage on these associations.

**Methods:**

Eighty-two participants aged 6 to 18 years (43.9% girls) from the Diactive-1 Cohort Study, diagnosed with type 1 diabetes, were included. Data collection involved continuous glucose monitoring, accelerometry to assess real-world PA, as well as documentation of daily insulin doses and carbohydrate counting over the same 7 days.

**Results:**

A total of 19 participants experienced at least 1 nocturnal hypoglycemia event over a span of 574 measurement days (106 days with and 451 days without nocturnal hypoglycemia). Higher levels of vigorous PA (VPA) were associated with lower same-day mean glucose levels (*P* = .014). Additionally, higher levels of moderate PA (*P* = .023), VPA (*P* = .011), and moderate-to-vigorous PA (*P* = .010) were associated with a greater number of nocturnal hypoglycemia events. Specifically, a significant association was identified between VPA and nocturnal hypoglycemia events when the daily insulin dose was at or above 1.04 units per kilogram of body weight per day (*P* = .016).

**Conclusion:**

Daily VPA is associated with glucose reductions, potentially leading to more hypoglycemic episodes, particularly when there is an excess of daily insulin. This highlights the need for careful insulin management in children and adolescents with type 1 diabetes engaging in VPA.

Type 1 diabetes is an autoimmune condition that elicits the destruction of pancreatic beta cells, resulting in a deficit of insulin production (1). According to the International Diabetes Federation, over 1.2 million children and adolescents worldwide are estimated to have type 1 diabetes (2). The American Diabetes Association and the International Society for Pediatric and Adolescent Diabetes promulgate guidelines that mandate a minimum of 60 minutes of moderate to vigorous physical activity (MVPA) daily for those below 20 years of age (3, 4). Research on children and adolescents with type 1 diabetes indicates that regular physical activity (PA) is associated with improved glycated hemoglobin (HbA1c) levels, reduced glycemic variability, decreased risk of long-term complications, and enhanced overall health (3-5). Despite these recommendations and benefits, many individuals in this population do not meet the recommended PA levels (5). Additionally, sedentary behavior, regardless of factors such as HbA1c levels and diabetes duration, is linked to poorer glycemic control (6).

Fear of hypoglycemia, particularly nocturnal hypoglycemia, acts as a significant barrier to PA engagement among children and adolescents with type 1 diabetes, with studies highlighting its predominant concern, especially in those exposed to nocturnal hypoglycemia associated with PA (7). Variations in PA regimens exert different effects on glycemic control and on the occurrence of hypoglycemia. For instance, Jaggers et al (8) observed that total minutes of moderate (MPA) and vigorous PA (VPA) predict nocturnal hypoglycemia events in adolescent athletes with type 1 diabetes, with VPA alone predicting event duration. Metcalf et al (9) noted that 30 minutes of MVPA per day among 14- to 20-year-olds increases hypoglycemia risk overnight and the following day. In line with these findings, another study involving younger children aged 2 to 17 years demonstrated that even short bursts of VPA can lead to extended periods of asymptomatic nocturnal hypoglycemia. Additionally, for each additional 60 minutes of PA, there seems to be a 60% to 80% increased likelihood of encountering hypoglycemia (10).

Despite significant findings, limitations in reviewed studies hinder stronger correlations. Primarily, small sample sizes (9) and solely adult participants (11) constrain generalizability. The inclusion of athlete children may also skew conclusions on PA impacts (8). Uncertainty persists regarding nocturnal hypoglycemia's association with PA levels vs moderating variables like excessive insulin dosing. Conflicting insulin dose recommendations (12, 13) complicate accurate dosing, influenced by factors such as age, sex, body mass index, and therapy mode (14). Addressing this complexity, our study aimed to analyze the relationship between PA parameters, mean glucose levels, and nocturnal hypoglycemic events over 7 days, seeking to estimate an insulin dose threshold for nocturnal hypoglycemia event prevention in children and adolescents with type 1 diabetes.

## Methods

### Subjects

The Diactive-1 Cohort Study is a cross-sectional study focusing on children and adolescents with type 1 diabetes residing in the Autonomous Community of Navarra, Spain. Recruitment of participants was conducted between May 2021 and February 2022 at the Pediatric Diabetes Unit of the University Hospital of Navarra (Spain). The eligibility criteria for this study included patients with an age range between 6 to 18 years diagnosed with type 1 diabetes, with a disease duration of more than 6 months, and who agreed to participate. Exclusion criteria involved any comorbidities that might deter involvement in PA (such as cardiovascular conditions, musculoskeletal disorders, or respiratory issues) or deficient understanding of the Spanish language. Out of the 183 patients under the care of the Pediatric Endocrinology Unit, 143 met the eligibility criteria, and 82 patients agreed to participate, resulting in a participation rate of 57.3%.

All subjects signed a written assent form, or their parents or legal guardian signed a written informed consent form before the study. Approval for the study was obtained from the Drug Research Ethics Committee of the University Hospital of Navarra (PI_2021/32), ensuring compliance with the principles outlined in the Declaration of Helsinki.

### Anthropometric and Body Composition Parameters

Height measurement was performed with individuals standing barefoot, ensuring their heels were together and touching the base of the vertical measuring column. Participants were guided to maintain a straight back and position their head in the Frankfurt horizontal plane (15). The SECA® 213 stadiometer from Hamburg, Germany, was used for measuring standing height, with the recorded values rounded to the nearest 0.1 cm. Body weight was measured in bare feet and lightweight clothing to the nearest 0.1 kg using a SECA® electronic scale (Scale 869). Body mass index was measured by dividing the weight in kilograms by the square of the height in meters. Finally, sitting height was measured using the SECA® 213 stadiometer and a wooden box.

### PA Levels and Sedentary Time

The GENEActiv® triaxial accelerometer (ActivInsights), worn on the nondominant-hand wrist, was utilized to measure the volume and intensity of PA. Accelerometers were programmed to measure at a frequency of 85.7 Hz across 7 consecutive days (16). The research group determined that sampling 86 times per second was enough to capture the greater part of movements executed by subjects. Accelerometer data were collected using GENEActiv® PC Software (version 3.3) and processed and analyzed using the R package GGIR (17). The eligible instances for waking wear time encompassed children and adolescents who logged a minimum of 10 hours of wear time while awake within a 24-hour period, including at least 1 weekend day. Validated cut-points were employed to ascertain the following PA variables (18-20): sedentary activity (for children: 0-56.3 milligravity [mg]; for adolescents: 0-50 mg), light PA (LIPA) (for children: 56.3-191.6 mg; for adolescents: 50-150 mg), MPA (for children: 191.6-695.8 mg; for adolescents: 150-500 mg), and VPA (for children: > 695.8 mg; for adolescents: > 500 mg). MVPA was defined as activities for which at least 80% of 1 minute of time satisfies the MPA threshold criteria (ie, 191.6 mg for children and 150 mg for adolescents), in order to remove signals related to random wrist movement (21).

### Peak Height Velocity

The peak height velocity (PHV), a common indicator of growth and development in children and adolescent (22), was obtained using anthropometric measures (weight, height, and seated height) as per Moore's equations (23). To calculate the years after PHV, we subtract the age at PHV from the actual age. The difference in years between these values is referred to as the maturity offset.

### Diabetes Assessment, Daily Insulin Dose and Carbohydrate Dose

Information regarding the therapy method (ie, multiple daily insulin injections or continuous subcutaneous insulin infusion via a pump system) and the duration of the illness was extracted from medical records. Participants kept a diary for 7 days, documenting their carbohydrate intake (1 serving = 10 g of carbohydrates) and the daily doses of insulin administered on the same days as when accelerometer data was collected. The diary relied on participant-reported data from diabetes devices, like insulin pumps or records of injections. The collected information was used to calculate the total carbohydrates (ration/day) and the insulin units per day per kilogram of body weight (units/kg/day).

### Average Glucose Level and Nocturnal Hypoglycemia

All of the participants used either the continuing glucose monitor (CGM) FreeStyle 2® Libre (Abbott Diabetes Care) or the MiniMed® 640G, 740G or 780G (Medtronic). These devices measured interstitial glucose levels every 60 seconds and generated glucose values every 15 minutes, along with corresponding glucose curves. The data collected was summarized in the ambulatory glucose profile report, which included the number of hypoglycemic events per day and the mean glucose level during this period. Nocturnal hypoglycemic events were characterized by the occurrence of 2 consecutive CGM readings below 70 mg/dL, with readings taken at 5-minute intervals between 8:00 Pm and 8:00 Am. An episode concluded when there were at least 2 CGM readings above 70 mg/dL.

### Statistical Analysis

The statistical analysis aimed to explore the association between PA, mean glucose levels, and nocturnal hypoglycemic events. Due to the nonnormal distribution of our data, the differences between groups were analyzed using a Mann–Whitney U test for continuous data and a chi-square test for categorical data.

Linear mixed-effects analysis was utilized to accommodate multiple outcome measures per participant. This analysis was developed with mean glucose and occurrences of nocturnal hypoglycemia as the dependent variables of the same day, while LIPA, MPA, VPA, sedentary time, and MVPA were considered as predictor variables. All models were adjusted for PHV, HbA1c levels, carbohydrate dosage, insulin pump usage, and duration of the disease. In addition, each PA parameter was adjusted for each other; for example, sedentary time was also adjusted for LIPA and MVPA.

Finally, moderation analyses were conducted using the PROCESS package. PROCESS employs ordinary least squares regression analysis to predict continuous variables such as attention capacity. Additionally, a bias-corrected bootstrap method with 5000 bootstrapped samples was utilized to estimate the conditional effects. The Johnson–Neyman technique was employed to determine whether daily insulin dose moderated the relationship between PA patterns and nocturnal hypoglycemia, thereby identifying regions of significance (24). In the context of the current study, the technique highlights the specific insulin dose at which the significant relationship between PA patterns and nocturnal hypoglycemia appears or disappears, as well as how that relationship varies based on the insulin dose. These analyses were adjusted for the aforementioned variables as well as the time of day during which MVPA was performed.

All analyses were performed in R (Version 4.3.2) and RStudio (Version 2023.09.1 + 494). Statistical significance was defined as 2-sided *P* < .05.

## Results

Table 1 displays the baseline characteristics of participants from the Diactive-1 Cohort Study, stratified by the occurrence of nocturnal hypoglycemia events. A total of 19 participants experienced at least 1 nocturnal hypoglycemia event over a span of 574 measurement days included in the analyses. Eight and 11 subjects in the nocturnal hypoglycemia group and 21 and 42 subjects in the no nocturnal hypoglycemia group used the Medtronic insulin pump or the FreeStyle Libre 2, respectively (*P* = .025). Significant differences were also observed between the 2 groups, particularly in HbA1c levels, average daily glucose levels, and PA patterns. We found no significant differences in demographic and clinical variables between study participants and dropouts. Additionally, none of the included subjects had issues such as retinopathy, nephropathy, or peripheral neuropathy.

**Table 1.** Presents the baseline characteristics of participants from the diactive-1 cohort study, comparing those with and without nocturnal hypoglycemia events

	With nocturnal hypoglycemia n = 19	Without nocturnal hypoglycemia n = 63	U—χ^2^ *	*P*
Age, years old	13 (11-15)	13 (11-15)	23 444.50	.757
Girls, n (%)	40.6	44.3	0.50	.480
Medtronic insulin pump, %	44.3	32.8	5.01	.025
FreeStyle Libre 2, %	55.7	67.2		
Duration of diabetes, years old	4.21 (2.31-7.31)	4.08 (2.00-7.00)	22 397.00	.312
Body mass index, kg/m^2^	19.86 (17.96-23.79)	19.21 (17.77-22.42)	21 469.50	.103
Glycated hemoglobin, %	7.10 (6.67-7.60)	7.40 (6.90-8.00)	19 397.50	.002
Peak high velocity, (score)	0.06 (−2.05-1.27)	−0.04 (−1.71-1.22)	23 407.50	.740
Carbohydrates, rations/day	16.50 (14.00-21.00)	16.80 (13.00-19.50)	17 593.00	.309
Insulin dose, Units/kg/day	0.76 (0.63-0.92)	0.72 (0.61-0.95)	17 986.50	.607
Periods of MVPA throughout the day				
Never, %	48.1	57.9	3.32	.069
Morning, %	0.9	4.0	2.42	.120
Afternoon, %	37.7	31.5	1.52	.217
Both morning and afternoon, %	13.2	6.7	3.41	.070
Average daily glucose levels, mg/dL	160.00 (138.00-186.25)	171.00 (146.75-200.25)	16 675.00	.005
Sedentary time, minutes per day	666.17 (575.66-752.49)	704.25 (627.08-778.06)	16 877.50	.020
Light-intensity physical activity, minutes per day	213.87 (185.75-259.27)	208.92 (166.00-257.50)	22 016.00	.206
Moderate-intensity physical activity, minutes per day	88.46 (63.83-119.02)	70.17 (45.42-99.92)	17 811.50	<.001
Vigorous-intensity physical activity, minutes per day	9.96 (3.73-22.08)	5.42 (1.50-14.75)	18 305.50	<.001
MVPA, minutes per day	39.04 (20.19-62.37)	23.92 (8.25-53.50)	17 747.00	<.001

Data are shown as median (interquartile range), n (%) or %.

Abbreviation: MVPA, moderate to vigorous physical activity.

^*^For continuous values, the Mann–Whitney U test was used, and for percentages, the chi-square test (χ^2^) was employed.

**Figure 1.**
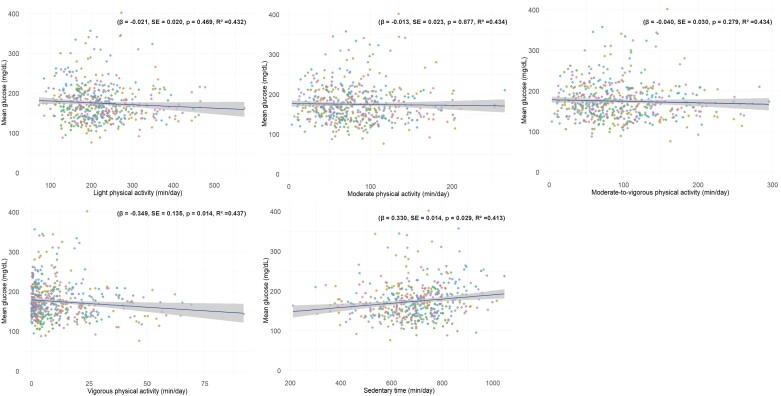
Association between light physical activity, moderate physical activity, vigorous physical activity, moderate to vigorous physical activity, and sedentary time with the mean glucose concentration over 7 days. Each participant is represented by a dot, with each dot repeating 7 times to signify measurements taken over 7 days. The line and shaded region illustrate the mean ± SEM across all participants.

Figure 1 elucidates the correlation between daily PA patterns and mean glucose levels. An inverse relationship was observed with VPA, where increased VPA was linked to reduced mean glucose levels on the same day (β = −.349, SE = 0.135, *P* = .014, R^2^ = 0.437). Conversely, a positive correlation was noted for sedentary durations, with longer inactivity correlating with higher mean glucose concentrations (β = .330, SE = 0.014, *P* = .029, R^2^ = 0.413). LIPA (β = −.021, SE = 0.020, *P* = .469, R^2^ = 0.432), MPA (β = −.013, SE = 0.023, *P* = .877, R^2^ = 0.434), and combined MVPA (β = −.040, SE = 0.030, *P* = .279, R^2^ = 0.434) showed no significant associations with mean glucose concentration.

**Figure 2.**
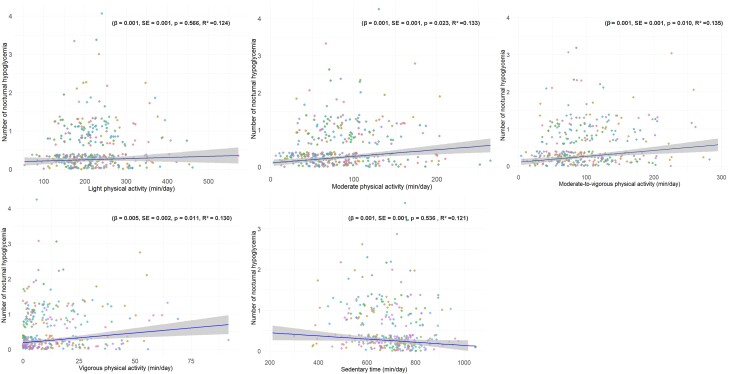
Association between light physical activity, moderate physical activity, vigorous physical activity, moderate to vigorous physical activity, and sedentary time with the incidence of nocturnal hypoglycemia events over 7 days. Each participant is represented by a dot, with each dot repeating 7 times to signify measurements taken over 7 days. The line and shaded region illustrate the mean ± SEM across all participants.

Regarding PA patterns’ influence on nocturnal hypoglycemic episodes, our analysis, illustrated in Fig. 2, establishes that elevated levels of MPA, VPA, and their combination (MVPA) are statistically correlated with an upsurge in nocturnal hypoglycemic events across the 7-day period. Specifically, this association is significant for MPA (β = .001, SE = 0.001, *P* = .023, R^2^ = 0.133) (Fig. 2A), VPA (β = .005, SE = 0.002, *P* = .011, R^2^ = 0.130), and MVPA (β = .001, SE = 0.010, *P* = .010, R^2^ = 0.135). Conversely, LIPA (β = .001, SE = 0.001, *P* = .566, R^2^ = 0.124) and sedentary time (β = .001, SE = 0.001, *P* = .536, R^2^ = 0.121) did not demonstrate a consequential relationship with these events.

**Figure 3.**
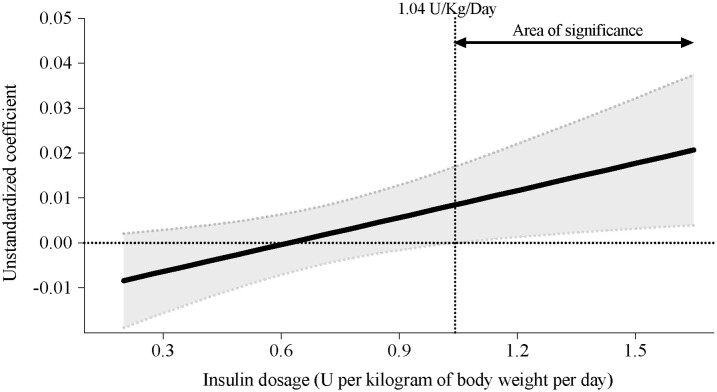
Moderation analysis examining the relationship between vigorous physical activity and the occurrence of nocturnal hypoglycemia, with insulin dose as the moderator, over a 7-day period.

Furthermore, applying the Johnson–Neyman statistical procedure identified a critical juncture at which insulin dosage may modulate these effects. As depicted in Fig. 3, a pivotal correlation was discerned between VPA and nocturnal hypoglycemia incidences at or above an insulin dose of 1.04 units/kg/day (*P* = .016). While a moderation effect was evident for MVPA, no discrete insulin dosing threshold was discernible.

## Discussion

Our findings suggest that engaging in VPA is associated with lower daytime blood glucose levels, while sedentary behavior is linked to higher levels in children and adolescents with type 1 diabetes. Both MVPA and VPA are also associated with increased nocturnal hypoglycemia events, but the daily insulin dose may influence these relationships. This implies that, given the numerous benefits of PA in children and adolescents, particularly VPA (25), it may be advisable to improve insulin management to mitigate the risk of hypoglycemic events.

While PA offers numerous benefits for children and adolescents with type 1 diabetes, it also presents a potential risk of hypoglycemia following activity, irrespective of its intensity. Our study has made a substantial contribution to elucidating this aspect. On one hand, an increase in MVPA and VPA were associated with a lower mean glucose concentration. In contrast, sedentarism is implicated in heightened mean glucose concentrations, resonating with the established correlation between prolonged inactivity, poor glycemic control (6), and augmented glucose levels in the absence of structured exercise (26, 27). These insights are echoed by research involving adults with type 1 diabetes, underscoring the link between sedentary behavior and increased glucose concentration (11). Possible reasons why some individuals have high levels of sedentary time could include loss of diabetes control and fear of hypoglycemia (28), identified as a major barrier preventing children and adolescents from engaging in PA and spending more time in sedentary activities (28-30). However, previous results observed in our cohort have shown that, paradoxically, children and adolescents with type 1 diabetes who fear hypoglycemia are more active and less sedentary than their counterparts who do not have this fear (31).

In light of the findings, our study delineates a distinct association between the intensity of PA—encompassing MPA, VPA, and MVPA—and the frequency of nocturnal hypoglycemia events. While MPA is associated with an increased number of these events, it alone does not significantly forecast the occurrence or duration of hypoglycemic episodes, a distinction that VPA markedly does (8). Regarding MVPA, this research has also shown a notable correlation between the overall amount of MVPA and the occurrence of nocturnal hypoglycemia (8). Research on the glycemic responses of children with type 1 diabetes to various PA intensities reveals an amplified risk of nocturnal hypoglycemia with MVPA sessions that extend beyond 1 hour (9, 10, 32). Moreover, even brief periods of intense PA can result in extended asymptomatic nocturnal hypoglycemia. For each additional 60 minutes of activity, there is a 60% to 80% greater risk of hypoglycemia (10). This heightened risk may be attributed to higher levels of PA potentially increasing insulin sensitivity and impacting the counterregulatory system, which could lead to inadequate insulin level control and an elevated risk of exercise-induced hypoglycemia (32-35). However, regarding nocturnal hypoglycemia associated with PA, our data indicate that patients who experience 1 or more episodes of hypoglycemia during this period exhibit lower levels of HbA1c. This suggests that promoting PA of a certain intensity may be important for achieving better glycemic control (6). Previous studies have shown an inverse association between hypoglycemia and HbA1c levels in youth with type 1 diabetes (36-39). This lower HbA1c level in subjects with nocturnal hypoglycemia reflects the delicate balance between achieving tight glycemic control and preventing hypoglycemic episodes. While lower HbA1c levels generally indicate better overall blood glucose management, in the context of PA, they may also signal an increased risk of nocturnal hypoglycemia (37). Our findings highlight the necessity of carefully monitoring blood glucose levels and adjusting insulin doses in active young individuals, particularly to manage nocturnal hypoglycemia due to potential delayed hypoglycemic events from PA (40). Educating patients and caregivers about this risk and appropriate preventive measures, such as bedtime carbohydrate intake, is crucial.

Another important aspect to consider is the timing of PA. It is important to highlight that our study includes as a covariate the time of day when young people engage in MVPA. Furthermore, we did not observe differences between different times of day and the presence or absence of hypoglycemia. This could indicate that, in our study, the time of day does not appear to be associated with a higher nocturnal hypoglycemia event. However, in contrast to our findings, earlier studies involving adolescents diagnosed with type 1 diabetes indicate that engaging in exercise during the late afternoon may result in delayed-onset hypoglycemia, especially following activities conducted during this time period (41). Furthermore, a study found that late-afternoon VPA increases postmeal but not overnight hypoglycemia in adults with type 1 diabetes, underscoring the significance of exercise timing in glycemic control among these patients (42).

Furthermore, we observed an association between VPA and the occurrence of nocturnal hypoglycemia when the insulin dose equaled or exceeded 1.04 units per kilogram of body weight per day. It is important to highlight that the number of subjects with insulin pumps among those who were above and below this cutoff point was not significant (*P* = .870, data not shown), suggesting that the insulin pump may not influence these findings. In this regard, Riddell et al (43) have shown in a study involving 251 Canadian adolescents and assessing acute glycemic responses to exercise that the magnitude of the drop in glucose was more pronounced when the concentration of glucose and insulin on board was high. This observation could partially explain our findings. Previous research by Kruszynska and Home (13) also suggested that individuals with type 1 diabetes traditionally required high insulin doses (>1.0 units per kilogram of body weight per day) to achieve satisfactory diabetic control. However, a statement from the American Diabetes Association (44) recommends reductions in basal insulin doses without specifying a cutoff point for how much the insulin dose can be reduced. Our findings indicate a potential cutoff point, which may be a significant factor contributing to these hypoglycemic events rather than VPA alone. In this regard, there are various recommendations regarding insulin and PA. For children and adolescents employing multiple daily injections, strategies include decreasing the daily basal insulin dose by around 20%, modifying prandial bolus insulin, and consuming low glycemic index carbohydrates after evening exercise (3). Similarly, those using continuous subcutaneous insulin infusion may find it beneficial to reduce basal rates by 20% at bedtime for 6 hours after afternoon exercise to mitigate the risk of nocturnal hypoglycemia (3). Finally, according to the age of the participants of our cohort study, adolescents, especially those with type 1 diabetes, experience significant hormonal fluctuations during puberty, impacting insulin sensitivity and metabolic responses (45, 46). Hormonal changes, such as increased levels of estrogen and testosterone, contribute to insulin resistance, potentially exacerbating glycemic control issues (47, 48). Therefore, managing insulin therapy based on pubertal stage becomes crucial to adjust to these physiological changes and optimize treatment outcomes.

### Strengths and Limitations

Strengths of the current study include the utilization of CGM to evaluate mean glucose levels and the frequency of nocturnal hypoglycemic events. Additionally, accelerometry was employed to assess patterns of PA in real-life settings, while participants maintained a diary documenting their insulin doses and carbohydrate intake over a 24-hour period for 7 consecutive days, providing a comprehensive understanding of their normal routines.

However, a limitation of the current study is that some patients experienced issues with their CGM devices (FreeStyle 2 and Medtronic), including surveillance range, accuracy, loss of sensitivity during bouts of hypoglycemia, and numerical errors (49, 50). Additionally, while CGM devices provide continuous glucose monitoring, they capture a subset of hypoglycemic events, including those with symptoms or prompted by alarms, which may lead to underreporting. Also, the use of different CGM devices could be another significant limitation. Finally, despite establishing a specific insulin cutoff point, determining a definitive association is challenging due to the availability of various types of insulin.

## Conclusion

We found that higher levels of MPA, VPA, and MVPA are associated with an increased risk of experiencing nocturnal hypoglycemia events. Moreover, when VPA levels are higher and the insulin dose equals or exceeds 1.04 U/kg/day, the likelihood of experiencing these events is even greater. Therefore, it is crucial for healthcare personnel to understand the risks of hypoglycemia that could occur in the pediatric population when engaging in VPA, particularly if insulin dosage exceeds recommended levels. Adherence to the recommendations provided by the Society for Pediatric and Adolescent Diabetes for children and adolescents with type 1 diabetes is essential in managing these risks (3) and adjusting insulin levels as appropriate.

## Data Availability

Some or all datasets generated during and/or analyzed during the current study are not publicly available but are available from the corresponding author on reasonable request.
